# Fructan Concentrations in Cooked Cereal Grains as a Nutritional Consideration for Low-FODMAP Diet

**DOI:** 10.3390/molecules29020282

**Published:** 2024-01-05

**Authors:** Ewa Pejcz, Agata Wojciechowicz-Budzisz, Radosław Spychaj, Zygmunt Gil, Joanna Harasym

**Affiliations:** 1 Department of Biotechnology and Food Analysis, Wroclaw University of Economics and Business, 53-345 Wroclaw, Poland; agata.wojciechowicz-budzisz@ue.wroc.pl; 2 Department of Fermentation and Cereals Technology, Wroclaw University of Environmental and Life Sciences, 51-630 Wroclaw, Poland; radoslaw.spychaj@upwr.edu.pl (R.S.); zygmunt.gil@upwr.edu.pl (Z.G.)

**Keywords:** nutritional value, cereals, fructans, FODMAPs, cooking, dietary guidance, low-FODMAP diet

## Abstract

Grains, essential for maintaining good health, contain short-chain carbohydrates like fructans, which can contribute to disorders in some individuals. Understanding and managing these FODMAPs (fermentable oligo-, di-, and monosaccharides and polyols) are essential for enhanced dietary guidance and well-being. The primary objective of the study was to establish safe portion sizes for grains and rice within low-FODMAP diets. A comprehensive analysis of fructan levels in diverse commercial cereal products contributes to an understanding of the potential digestive impact of FODMAPs in grains and supporting enhanced dietary guidance for individuals with FODMAP-related disorders. Various grains, like white and brown rice, barley, wheat groats, and buckwheat, highlight the challenges of handling fructans in a low-FODMAP diet. Fructans to heat-induced degradation, as demonstrated in bulgur, emphasize the need to consider cooking methods for managing their intake. Identification of potentially safe grains, like white long-grain rice and arborio rice, is significant, but caution is advised with barley groats and couscous, stressing personalized dietary decisions. Correlation analyses linking color parameters, moisture content, and fructan levels in cooked grains reveal a positive relationship, suggesting water content’s potential impact on fructan stability and grain hydration properties. In conclusion, the study provides valuable insights into the intricate details of FODMAPs in grains, supporting the development of dietary strategies that enhance both health and sensory satisfaction.

## 1. Introduction

Grains serve as a primary source of carbohydrates in the daily human diet. Apart from providing energy, carbohydrates from grains have a significant impact on various physiological processes in the human body, contributing to the prevention of numerous diseases. Carbohydrates can be categorized into short-chain carbohydrates (monosaccharides, oligosaccharides, and disaccharides) and long-chain carbohydrates (starch, resistant starch, and non-starch polysaccharides). The consumption of long-chain carbohydrates offers various benefits, including stool bulking, faster colonic transit time, and mild intestinal acidification [1,2]. Some short-chain carbohydrates, such as fructans (fructooligosaccharides and inulin) and galactooligosaccharides, are also known as prebiotics. Research has shown that they stimulate the growth and activity of beneficial colon bacteria, including bifidobacteria and lactobacilli. As a result, they are associated with many health benefits, including a reduced risk of gastrointestinal infections, improved metabolism, enhanced calcium absorption, stimulation of the gastrointestinal immune system, reduced risk of colorectal cancer, and lower blood glucose and cholesterol levels [2,3,4,5,6].

Despite the numerous health benefits of consuming short-chain carbohydrates, there is a segment of the population that cannot tolerate them, leading to a range of discomforting symptoms that significantly impact their quality of life [1]. These symptoms include irritable bowel syndrome (IBS), small intestinal bacterial overgrowth (SIBO), inflammatory bowel disease (IBD), and non-celiac gluten sensitivity (NCGS). Common functional gastrointestinal disorder symptoms include abdominal pain, bloating, and alterations in bowel habits (constipation, diarrhea, or mixed). These disorders can affect individuals of any gender, age, race, skin color, religion, or socio-economic status [7]. Worldwide, approximately 1 in 10 people suffer from IBS, with the majority residing in developed countries. This increase is likely due to an unhealthy diet, particularly one rich in highly processed products. Irritable bowel syndrome is characterized by recurring abdominal pain and changes in bowel habits (constipation, diarrhea, or a combination of both). IBS is diagnosed when symptoms have been present for six months before diagnosis and have occurred regularly in the last three months [7,8,9]. SIBO affects 2.5–22% of people worldwide and is characterized by an overgrowth of bacteria in the small intestine (over 10^5 bacteria per 1 mL of intestinal contents). It is often associated with other conditions, such as IBS, Crohn’s disease, celiac disease, short bowel syndrome, liver cirrhosis, non-alcoholic fatty liver disease, and neurological disorders. Symptoms of bacterial overgrowth are diverse and may include fat malabsorption, diarrhea, osmotic diarrhea, flatulence, and bloating. Non-gastrointestinal symptoms can also occur, including joint inflammation, kidney inflammation, and nodular erythema [10,11]. In the second half of the 20th century, North America and Europe saw a significant increase in the incidence of inflammatory bowel diseases. Alarmingly, this rise has also been observed in countries like Japan and India, previously considered low-risk areas. The primary causes of this increase are Western lifestyles, improper diets, and habits such as smoking. IBD includes Crohn’s disease and ulcerative colitis, both of which are immune-mediated diseases [12]. Non-celiac gluten sensitivity is a condition in which patients experience symptoms such as diarrhea or constipation, abdominal pain, bloating, anemia, muscle and joint pain, numbness of the extremities, sleep disturbances, chronic fatigue, and even depression. The prevalence of non-celiac gluten sensitivity in the general population is unknown as it is most often self-diagnosed by patients who experience improvement on a gluten-free diet. Physicians typically diagnose it after ruling out celiac disease and wheat allergies [13].

These conditions are attributed to FODMAPs, which stands for fermentable oligo-, di-, and monosaccharides and polyols. FODMAPs include lactose, excess fructose compared to glucose, polyols, fructans, and galactooligosaccharides [1,14,15]. The accumulation of FODMAPs in the small intestine results in increased osmotic pressure and increased liquid content in the intestines. Among the FODMAP compounds found in grain products, fructans are the most abundant. These are linear or branched fructose polymers, including short-chain fructans (fructooligosaccharides) and long-chain fructans (inulin). Their intolerance is mainly attributed to the lack of intestinal and pancreatic enzymes that break the β-glycosidic bond between two glucose molecules, leading to reduced absorption, and undigested fructans that ferment in the colon [14]. Additionally, grains may contain little amounts of galactooligosaccharides, primarily raffinose, and, in small or trace amounts, stachyose. No other significant FODMAPs are present in grains, with very small amounts of fructose not exceeding glucose content and polyols (sorbitol and mannitol) at 0.01–0.04 g/100 g dry weight [16].

The treatment of IBS, IBD, and other FODMAP-related conditions often involves dietary changes. One of the most widely recognized dietary approaches is the low-FODMAP diet, which restricts the intake of fermentable carbohydrates. Reducing FODMAPs in the diet can help alleviate symptoms for many individuals with these disorders. The low-FODMAP diet typically consists of three stages: restriction, reintroduction, and personalization [17]. In the restriction phase, high-FODMAP foods are avoided, and patients typically experience symptom relief. Despite its effectiveness, the low-FODMAP diet poses several challenges. The most significant challenge is its restrictive nature as it eliminates many common and nutritious foods. This restriction can lead to nutritional deficiencies, particularly in dietary fiber and important prebiotic components [17]. The diet also requires careful planning and monitoring to ensure it meets an individual’s nutritional needs. Additionally, adhering to the low-FODMAP diet can be socially and emotionally challenging due to limited food choices and potential stigmatization.

The main objective of this study was to evaluate and establish the safe portion sizes of grains and rice for individuals following a low-FODMAP diet by determining the fructans content in various commercial cereal products from diverse origins, bringing a scientific lens to the assessment. The limit values indicated by Varney et al. (2017) [15] for the content of fructans in a portion of the product were adopted at the level of 0.3 g in a portion of the cooked product. In this pursuit, it was recognized that grains, while providing essential carbohydrates and nutrients, can also contain FODMAPs, potentially triggering digestive issues in susceptible individuals. Furthermore, this study aimed to contribute not only to the ongoing development of the low-FODMAP diet but also introduce innovative approaches to providing effective dietary guidance. By unraveling the complexities surrounding FODMAPs in grains and rice, this study aimed to enhance the quality of life for individuals dealing with FODMAP-related disorders.

## 2. Results

### 2.1. Water Absorption of Groats

Comparison among the moisture content of cooked grains reveals significant variations across different grain types (Table 1). Notably, parboiled rice exhibited the highest moisture content at 72.91%, while sushi rice demonstrated the lowest at 59.83%. Brown rice, basmati rice, and jasmine rice fell within an intermediate range. In the wheat groats category, semolina displayed the highest moisture content (77.16%), contrasting with bulgur wheat (59.53%). Among barley groats, fine pearl barley groats recorded the highest moisture content (76.93%), followed by country-style barley groats and barley groats. In the broader spectrum, corn groats exhibited the highest moisture content at 88.97%, surpassing both roasted buckwheat groats and white buckwheat groats. These findings offer insights for nutritional considerations, particularly in terms of portion sizes for carbohydrate intake, as moisture content can influence the overall weight of a given portion of cooked grains.

### 2.2. Color Parameters in Freshly Cooked Groats

The color characteristics of various cooked grains are detailed in Table 2, where L*, a*, and b* values represent the lightness, greenness to redness, and blueness to yellowness, respectively. Among the rice varieties, jasmine rice displayed the highest lightness (L* = 79.90), while brown rice exhibited the lowest (L* = 67.10). Basmati rice demonstrated the highest b* value (yellowness), and sushi rice had the lowest a* value (greenness). In the wheat groats category, semolina exhibited the highest lightness (L* = 78.70) and the most negative a* value (greenness). Couscous presented the highest b* value (yellowness) among wheat groats. Barley groats showed variations in lightness, with fine pearl barley groats having the highest L* value (63.10). Among others, corn groats displayed the highest b* value (45.70), indicating a pronounced yellowness. Roasted buckwheat groats exhibited the lowest L* value (46.50), signifying a darker appearance.

### 2.3. Fructan Content in Cooked Cereal Grains

The fructan content in the dry mass of the examined grains underscores notable differences across various grain types (Table 3). Notably, couscous demonstrated the highest fructan content at 2.04 g/100 g, followed by fine pearl barley groats (1.44 g/100 g) and semolina (0.83 g/100 g). Barley groats and country-style barley groats presented moderate fructan content at 0.59 g/100 g and 0.80 g/100 g, respectively. Among rice varieties, brown rice exhibited the highest fructan content (0.26 g/100 g), while jasmine rice, arborio rice, and white long-grain rice showed relatively lower fructan content. Corn groats displayed the highest fructan content among other gluten-free grains at 0.37 g/100 g. Roasted buckwheat groats and white buckwheat groats demonstrated similar fructan content. The results provide insights into the fructan composition of various grains, offering information for dietary planning, particularly for individuals who need to monitor fructan intake due to dietary restrictions or sensitivities.

**Figure 1:**
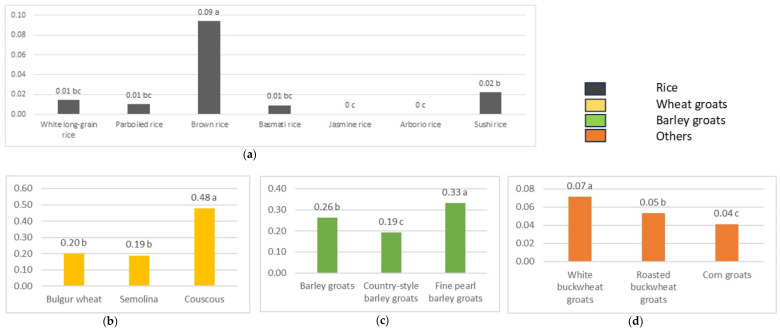
Fructan content in cooked cereal grains [g/100 g]. (**a**) Rice; (**b**) Wheat groats; (**c**) Barley groats; (**d**) Others. Values represent means, lowercase letters indicate homogeneous groups determined using the Duncan test.

The fructan content in various freshly cooked cereal grains, classified into (a) Rice, (b) Wheat groats, (c) Barley groats, and (d) Others is shown in Figure 1. In the Rice category, brown rice exhibits the highest fructan content at 0.09 g/100 g, distinguishing itself from other rice varieties—white long-grain rice, parboiled rice, basmati rice, jasmine rice, arborio rice, and sushi rice—which demonstrate lower and comparable fructan contents. Within Wheat groats, couscous stands out with the highest fructan content at 0.48 g/100 g, surpassing semolina and bulgur wheat, both displaying lower fructan content. In the Barley groats category, fine pearl barley groats lead with the highest fructan content at 0.33 g/100 g, while barley groats and country-style barley groats exhibit comparatively lower fructan content. The Others category reveals that corn groats have the highest fructan content at 0.04 g/100 g, while white buckwheat groats and roasted buckwheat groats display lower and similar fructan content.

**Figure 2:**
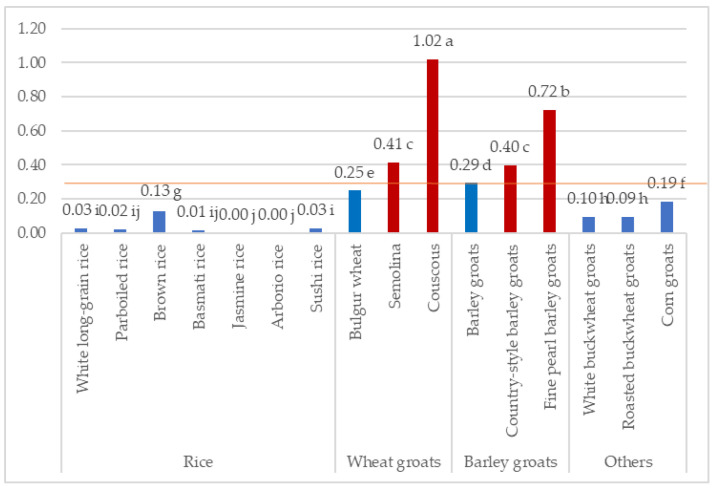
Fructan content per serving of the product [g]: blue bars illustate values below the intake threshold, red bars- values above the intake threshold. Values represent means, lowercase letters indicate homogeneous groups determined using the Duncan test.

The fructan content in a 50 g portion of various cooked grains, representing a typical serving size before cooking, is detailed Figure 2, with cut-off values of 0.3 g per serving considered significant for a low-FODMAP diet. Notably, couscous surpasses the cut-off, containing 1.02 g of fructans per serving, suggesting a higher fructan content. Conversely, other grains, including white long-grain rice, parboiled rice, basmati rice, jasmine rice, arborio rice, sushi rice, white buckwheat groats, roasted buckwheat groats, and corn groats, exhibit fructan levels below the 0.3 g threshold, making them potentially suitable for individuals following a low-FODMAP diet. The results show the need for careful selection of grains to meet dietary restrictions associated with fructan intake.

### 2.4. Correlation between Fructan Content and Color Parameters of Groats

Table 4 displays Pearson’s correlation coefficients (significance level α ≤ 0.05) among key parameters in cooked cereal grains. The moisture content (MC) exhibits a significant (marked in red) positive correlation with fructan content in the dry mass of the product (FDM) at 0.43, indicating a relationship between moisture content and fructan levels. The lightness parameter (L*) demonstrates a strong negative correlation with a* color parameter (−0.95), suggesting that as yellowness increases, the lightness of the grains decreases. Moreover, the lightness of grains is negatively correlated with fructan content in both dry mass and cooked cereal grains. The a* parameter displays a positive correlation with fructan levels in freshly cooked grains (0.44), while b* shows positive correlations with both fructan content in dry mass of grains (0.66) and freshly cooked grains (0.61). Fructan content in dry mass exhibits a strong positive correlation with fructan concentrations in freshly cooked grains (0.96). The remaining correlations (marked in black) were not statistically significant at α ≤ 0.05.

## 3. Discussion

The variations in moisture content observed among different cooked grains can be attributed to intrinsic characteristics, cooking methods, and varietal distinctions. Parboiled rice, with the highest moisture content among rice varieties at 72.91%, reflects its natural water absorption capacity and the parboiling process. Conversely, sushi rice, with the lowest moisture content at 59.83%, may undergo a cooking process that limits water absorption. Intermediate moisture levels in brown rice, basmati rice, and jasmine rice suggest varietal and inherent grain characteristics influencing water absorption during cooking. Granular structure, cooking duration, and pre-processing techniques contribute to these differences, as seen in parboiled rice. Corn groats exhibit the highest moisture content at 88.97%, highlighting a significant water absorption capacity. These findings underscore the importance of precise grain selection based on water absorption, critical for managing FODMAP intake on a low-FODMAP diet. Tailoring grain choices considering individual tolerances and dietary preferences is vital for optimizing satiety, portion size, and carbohydrate intake within diet parameters. The studies by Biesiekierski et al. (2011) [1] and Haskå et al. (2008) [18] complement these results, providing insights into fermentable short-chain carbohydrates like fructans, emphasizing the need for comprehensive composition tables to understand the intricate interplay between different components in grains and their potential physiological impacts on the gut.

The investigation into varying fructan levels in white and brown rice emphasizes the imperative for ongoing exploration, particularly in light of potential gluten contamination and the bran component in brown rice. The divergent fructan profiles between these rice varieties highlight the relationship between grain composition and fructan content, insisting on the understanding in the context of dietary management [1,19]. In the context of barley and wheat groats, the significant presence of fructans, particularly in items like couscous and bulgur, was noticed. This agreement emphasizes how these discoveries apply to various grains, adding to the increasing evidence that highlights the importance of thinking about fructan levels when planning diets [20,21]. The vulnerability of fructans to degradation under heating conditions potentially explains the comparatively lower fructan levels observed in bulgur, showing the impact of processing methods on the fructan composition of grains. Understanding such complexities is crucial for informed dietary choices [22,23]. The results of Nemeth et al. (2014) [24] show that a substantial fructan content in barley products underscores the influence of factors such as cultivation practices and plant varieties on fructan concentrations, emphasizing the need for a comprehensive evaluation of fructans in different grains with due consideration for agricultural practices. Incorporating the threshold values for fructan content proposed by Varney et al. (2017) [15] into the framework of a low-FODMAP diet offers a pragmatic approach to dietary management. Among the analyzed grains, white long-grain rice, arborio rice, and white buckwheat groats stand out as potential safe choices for individuals adhering to low-FODMAP diets. Nevertheless, it is essential to exercise caution when considering barley groats and couscous, underscoring the importance of personalized dietary decisions aligned with individual tolerances and preferences.

The color characteristics of cooked grains, as indicated by L*, a*, and b* values, not only provide visual appeal but also offer valuable insights into the nutritional composition and processing methods of these grains. Variations in lightness (L*) among different rice varieties, such as the lighter jasmine rice compared to the darker brown rice, may signify distinctions in nutritional content or processing techniques, potentially influenced by the presence of bran in brown rice, contributing to its lower lightness due to dietary fiber and other compounds. Wheat groats, exemplified by semolina’s high lightness and negative greenness (a*) value, may indicate variations in processing or refining, impacting their nutritional profile. The highest yellowness (b* value) in couscous among wheat groats raises questions about the compounds influencing this color attribute and its nutritional implications [25]. Correlation analyses reveal connections between color parameters, moisture content, and fructan levels in cooked grains. The positive correlation between moisture content (MC) and fructan content in the dry mass (FDM) during processing suggests a potential impact of water content on fructan stability or concentration, influenced by the hydration properties of grains and their interaction with fructans [26]. The negative correlation between L* and a* values indicate that as yellowness increases, the lightness of grains decreases, aligns with visual expectations, and may be attributed to pigments or compounds contributing to both color attributes. The positive correlation between a* values and fructan levels in freshly cooked grains hints at a potential link between the greenness of grains and fructan content, revealing a new relationship regarding the nature of these compounds. Moreover, positive correlations between yellowness (b* values) and fructan content in both dry mass and freshly cooked grains suggest that grains with a more pronounced yellowness may conceal higher fructan concentrations, emphasizing the need to explore compounds responsible for yellowness to gain insights into factors influencing fructan content [26,27]. The positive correlation between the greenness parameter (a*) and fructan levels in freshly cooked grains further suggests a potential link between the greenness of grains and fructan content. Integrating these color insights with the broader context of developing nutritionally rich yet sensorily appealing foods, especially considering the potential role of fructans and challenges related to FODMAPs, underscores the complexity and importance of innovative approaches in food science [28]. The moisture content of cooked grains, as indicated by the data, plays a crucial role in nutritional considerations for individuals adhering to a low-FODMAP diet. Understanding the variations across different grain types can help in managing portion sizes for carbohydrate intake as moisture content influences the overall weight of a given portion of cooked grains. Additionally, the fructan concentration highlighted in the findings underscores the importance of selecting grains wisely for those with dietary restrictions or sensitivities. Notably, couscous surpasses the cut-off for a low-FODMAP diet, emphasizing the need for careful consideration when choosing grains to meet specific dietary requirements associated with fructan intake and overall human health. Tailoring grain choices to adhere to a low-FODMAP diet involves strategic selection based on fructan levels. Grains like white long-grain rice, parboiled rice, and basmati rice consistently exhibit fructan levels below the 0.3 g threshold per serving, making them favorable choices for individuals following a low-FODMAP diet. Conversely, couscous stands out with a higher fructan content of 1.02 g per serving, surpassing the cut-off and suggesting caution for those with fructan-related dietary restrictions. Similarly, roasted buckwheat groats and white buckwheat groats demonstrate lower and comparable fructan content, making them potentially suitable options for individuals aiming to manage their FODMAP intake. These specific recommendations enable individuals to make informed choices, ensuring compliance with low-FODMAP guidelines while maintaining a varied and satisfying diet.

## 4. Materials and Methods

### 4.1. Materials

The material constituted commercially available cereal groats of different origins and pre-treatment. The details such as country of origin, manufacturer or distributor information, and recommended cooking times are presented in Table 5.

### 4.2. Sample Preparation

The cereal groats underwent controlled cooking by immersing them in excess water, following precise instructions provided by manufacturers: Sawex Foods, Lestello, Kupiec, Sante, and Cenos. This approach ensured optimal hydration, gelatinization, and adherence to specific texture guidelines. The cooking times, ranging from 3 to 35 min as detailed in Table 5, align with industry standards. Draining was executed to halt the process, resulting in final products with defined physical and chemical properties. It is noteworthy that samples of each product were prepared twice to enhance the reliability and reproducibility of the obtained data. Researchers seeking additional information on grain handling can refer to the manufacturers’ recommendations for these specific groats.

### 4.3. Water Absorption of Groats

The assessment of water absorption in the cooked groats was performed using the drying method, with a primary focus on the quantification of moisture content. The quantification of moisture content was executed using the formula:

Moisture (%) = (initial sample weight − weight of dried sample)/(initial sample weight) × 100%.

Through the application of this method, a measurement and calculation were conducted to determine the percentage of moisture retained in the groats post-cooking, providing data regarding the efficiency of water absorption during the cooking process. The measurement for each batch was performed in duplicate.

### 4.4. Cooked Grains Color

The color of the cooked groats was evaluated using the L*, a*, and b* parameters measured with a Konica Minolta Chroma Meter CR-410 reflective colorimeter (Konica Minolta, Tokyo, Japan). A luminosity parameter, L*, registering at 0 denotes black, while a value of 100 signifies white. The a* and b* parameters exhibit both positive and negative values, each connoting specific color attributes. The negative a* parameter corresponds to the green spectrum, while its positive counterpart, a*+, signifies red. Additionally, the b*− parameter is indicative of yellow, while the positive b*+ parameter represents the color blue. The assessment involved measurements at ten different points on each batch of cooked cereal grains, with subsequent analysis of the calculated means.

### 4.5. Fructans Content

Freshly cooked and freeze-dried groat samples underwent fructan content analysis using the enzymatic-fotometric method (AOAC Method 999.03). This involved determining the fructose content in samples resulting from the enzymatic breakdown of fructans using a fructan measurement kit (Magazyme, Brey, Ireland). Absorbance was measured in a spectrophotometer (GENESYS 10S, Thermo Fisher Scientific, Waltham, MA, USA) at a wavelength of 410 nm using standard spectrophotometric cuvettes PS 4.5 mL. The measurement for each batch was performed in duplicate.

### 4.6. Statistical Analysis

The results were processed using Statistica 13.3. One-way analysis of variance (ANOVA) was employed, with the type of product as the factor. Duncan’s test was applied to identify differences between means at a significance level of α ≤ 0.05, and homogeneous groups were determined based on this test. Pearson’s correlation coefficients were calculated with a significance level of α ≤ 0.05.

## 5. Conclusions

Exploration into varying fructan levels in different grains, particularly in white and brown rice, barley, wheat groats, and buckwheat, highlights the complexity of managing fructans in the context of a low-FODMAP diet. Variability in fructan profiles among rice varieties, substantial presence in barley and wheat groats, and the observed vulnerability of fructans to degradation under heating conditions, particularly in bulgur, emphasize the ongoing need for comprehensive research to inform dietary choices. Identifying specific grains like white long-grain rice, arborio rice, and white buckwheat groats as potentially safe options for low-FODMAP diets is a significant outcome. However, caution is advised with barley groats and couscous, underscoring the importance of personalized dietary decisions based on individual tolerances and preferences. Correlation analyses, linking color parameters, moisture content, and fructan levels in cooked grains, reveal a positive correlation between moisture content and fructan levels during processing, suggesting the potential influence of water content on fructan stability and grain hydration properties. Understanding these nuances is crucial for making well-informed dietary choices that strike a balance between nutritional richness and sensory appeal within the constraints of a low-FODMAP diet.

## Figures and Tables

**Table 1:** Moisture content of cooked grains [%].

Grains	Moisture Content [%]
Rice	
White long-grain rice	70.66 ± 0.34 b
Parboiled rice	72.91 ± 0.09 a
Brown rice	63.15 ± 0.14 d
Basmati rice	62.67 ± 0.33 de
Jasmine rice	65.52 ± 0.31 c
Arborio rice	61.71 ± 0.29 e
Sushi rice	59.83 ± 0.17 f
Wheat groats	
Bulgur wheat	59.53 ± 0.47 b
Semolina	77.16 ± 0.16 a
Couscous	76.57 ± 0.36 a
Barley groats	
Barley groats	55.17 ± 0.17 c
Country-style barley groats	75.82 ± 0.18 b
Fine pearl barley groats	76.93 ± 0.08 a
Others	
White buckwheat groats	71.29 ± 0.28 b
Roasted buckwheat groats	62.33 ± 0.33 c
Corn groats	88.97 ± 0.04 a

Values represent means ± standard deviation. Small letters indicate homogeneous groups determined using the Duncan test (*p* ≤ 0.05).

**Table 2:** Cooked grains color.

Group	Grains	L*	a*	b*	Visualization
Rice	White long-grain rice	77.85 b	−3.85 d	12.05 f	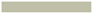
	Parboiled rice	75.25 c	−2.95 b	14.70 d	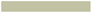
	Brown rice	67.10 d	−0.55 a	18.80 a	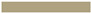
	Basmati rice	78.05 b	−3.05 b	13.95 e	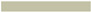
	Jasmine rice	79.90 a	−4.25 e	14.15 e	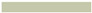
	Arborio rice	77.55 b	−3.55 c	15.55 b	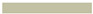
	Sushi rice	79.70 a	−4.45 f	15.15 c	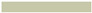
Wheat groats	Bulgur wheat	66.95 c	−1.95 a	28.70 b	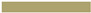
	Semolina	78.70 a	−6.50 c	17.90 c	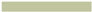
	Couscous	74.75 b	−3.05 b	29.80 a	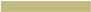
Barley groats	Barley groats	57.85 c	1.85 a	18.95 a	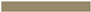
	Country-style barley groats	62.15 b	0.45 a	17.75 b	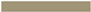
	Fine pearl barley groats	63.10 a	0.90 a	18.05 b	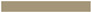
Others	White buckwheat groats	54.90 b	2.30 b	13.55 b	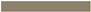
	Roasted buckwheat groats	46.50 c	5.05 a	13.95 b	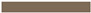
	Corn groats	75.80 a	−3.90 c	45.70 a	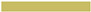

Values represent means of 5 replicates. Small letters indicate homogeneous groups determined using the Duncan test (*p* ≤ 0.05).

**Table 3:** Fructans content in the dry mass of the product [g/100 g].

Grains	Fructan Content [g/100 g]
Rice	
White long-grain rice	0.05 ± 0.04 b
Parboiled rice	0.04 ± 0.01 b
Brown rice	0.26 ± 0.01 a
Basmati rice	0.03 ± 0.01 b
Jasmine rice	0.00 ± 0.00 b
Arborio rice	0.00 ± 0.00 b
Sushi rice	0.06 ± 0.01 b
Wheat groats	
Bulgur wheat	0.50 ± 0.01 c
Semolina	0.83 ± 0.03 b
Couscous	2.04 ± 0.01 a
Barley groats	
Barley groats	0.59 ± 0.02 c
Country-style barley groats	0.80 ± 0.01 b
Fine pearl barley groats	1.44 ± 0.01 a
Others	
White buckwheat groats	0.19 ± 0.01 b
Roasted buckwheat groats	0.19 ± 0.00 b
Corn groats	0.37 ± 0.01 a

Values represent means ± standard deviation. Lowercase letters indicate homogeneous groups determined using the Duncan test.

**Table 4:** Pearson’s correlation coefficients (significance level α ≤ 0.05).

	MC	L*	a*	b*	FDM	FFC
MC	1.00	0.06	−0.19	0.19	0.43	0.21
L*		1.00	−0.95	−0.13	−0.44	−0.56
a*			1.00	−0.03	0.28	0.44
b*				1.00	0.66	0.61
FDM					1.00	0.96
FFC						1.00

MC—moisture content; L*, a*, b*—color parameters; FDM—fructan content in the dry mass of the product; FFC—fructan content in fresh cooked cereal grains. Values marked in red indicate significant correlation coefficients at α ≤ 0.05.

**Table 5:** Country of origin and recommended cooking time for cereal groats.

Group	Grains	Country of Origin	Manufacturer/Distributor	Cooking Time [min]
Rice	White long-grain rice	Myanmar	Sawex Foods, Warsaw, Poland	14
	Parboiled rice	Thailand	Lestello, Cmolas, Poland	12
	Brown rice	Vietnam	Sawex Foods, Warsaw, Poland	35
	Basmati rice	India	Lestello, Cmolas, Poland	15
	Jasmine rice	Thailand	Lidl, Tarnowo Podgórne, Poland	12
	Arborio rice	Italy	Lidl, Tarnowo Podgórne, Poland	15
	Sushi rice	Japan	Lidl, Tarnowo Podgórne, Poland	10
Wheat groats	Bulgur wheat	Türkiye	Lidl, Tarnowo Podgórne, Poland	15
	Semolina	Poland	Kupiec, Krzymow, Poland	3
	Couscous	Italy	Sante, Warsaw, Poland	5
Barley groats	Barley groats	Poland	Cenos, Września, Poland	15
	Country-style barley groats	Poland	Cenos, Września, Poland	15
	Fine pearl barley groats	Poland	Kupiec, Krzymow, Poland	15
Others	White buckwheat groats	Poland	Sante, Warsaw, Poland	15
	Roasted buckwheat groats	Poland	Sante, Warsaw, Poland	15
	Corn groats	Poland	Sante, Warsaw, Poland	10

## Data Availability

The datasets used and analyzed during the current study are available from the corresponding author on reasonable request.
